# Major gut microbiota perturbations in firstborn infants compared to those with older siblings soon after delivery

**DOI:** 10.1186/s12887-025-06015-7

**Published:** 2025-10-07

**Authors:** Annika Ljung, Monica Gio-Batta, Bill Hesselmar, Henrik Imberg, Hardis Rabe, Forough L. Nowrouzian, Susanne Johansen, Agnes E. Wold, Ingegerd Adlerberth

**Affiliations:** 1 Department of Infectious Diseases, Institute of Biomedicine, Sahlgrenska Academy, University of Gothenburg, 405 30 Gothenburg, Sweden; 2 Institute of Clinical Sciences, Department of Paediatrics, Sahlgrenska Academy, University of Gothenburg, Gothenburg, Sweden; 3 Statistiska Konsultgruppen Sweden, Gothenburg, Sweden; 4 Department of Molecular and Clinical Medicine, Institute of Medicine, Sahlgrenska Academy, University of Gothenburg, Gothenburg, Sweden; 5 Pediatric Clinic, Skaraborg Hospital, Lidköping, Sweden

**Keywords:** Epidemiology, Infant, Microbiome, Older siblings, Cesarean section, Antibiotic exposure during delivery

## Abstract

**Background:**

The gut microbiota may influence immune maturation during infancy. While cesarean delivery is known to delay acquisition of a mature anaerobic microbiota, the influence of being firstborn on early gut colonization is less well studied.

**Methods:**

Feces were collected regularly from 3 days to 18 months of age in the FARMFLORA cohort (*N* = 65) and cultured quantitatively for major anaerobic and facultative bacteria. Colonization rates and population counts of different gut bacteria were analyzed in relation to birth order, delivery mode, and antibiotic exposure during delivery. Each exposure was adjusted for the other two.

**Results:**

Birth order, delivery mode and antibiotic exposure were each independently associated with gut colonization. Firstborn infants (*N* = 29) acquired *Escherichia coli* and bifidobacteria later than infants with older sibings (*N* = 36) and had signs of microbiota immaturity, including higher levels of facultative bacteria and increased carriage of opportunistic colonizers such as *Clostridioides difficile* and *Staphylococcus aureus*. Cesarean-delivered infants (*N* = 10) showed delayed *E. coli* acquisition and increased colonization by other enterobacteria, while antibiotic exposure during delivery (*N* = 10) delayed colonization by *Bacteroides*.

**Conclusions:**

Delayed colonization by typical fecal bacteria in firstborn infants suggests reduced maternal bacterial transfer during delivery. This might contribute to the predilection of firstborn children to develop allergy.

**Supplementary Information:**

The online version contains supplementary material available at 10.1186/s12887-025-06015-7.

## Background

The hygiene hypothesis for allergy development was proposed by Strachan in 1989 based on his discovery that firstborn and only children had a higher rate of allergic disease at 16 years of age than those who had older siblings [1]. As poverty had previously been linked to protection from allergy, Strachan proposed that the circulation of childhood infections, common in both large and poor families, in some way would educate the immature immune system, preventing it from later overreacting to harmless proteins in the food and air. Since then, a number of studies have confirmed the positive association between being firstborn and risk of allergy development [2], but the mechanism remains elusive.

Part of the explanation for the increased risk of allergy in firstborn children could be delayed establishment of the gut microbiota. In accordance, a gut microbiota of low complexity in the first weeks of life is associated with increased risk of later allergy [3–5]. Delayed maturation of the microbiota has also been linked to increased risk of allergy development [6, 7]. Gut microbiota maturation implies that the microbiota becomes successively more complex and dominated by anaerobes, a process that takes place over the first years of life [8, 9]. Directly after birth, the infant gut is oxygen-rich and bacteria that thrive in such a milieu, including *E. coli* and other members of the *Enterobacteriaceae* family, enterococci, staphylococci and streptococci, as well as aerotolerant anaerobes such as bifidobacteria and lactobacilli, establish and occupy the available ecological space. As oxygen is consumed, obligate anaerobes can survive and multiply. They expand into the ecological space of the facultatives, whose populations contract [8, 10]. *Clostridioides difficile*, a spore forming anaerobe, is also suppressed by a mature microbiota and is common in young infants, but disappears with time [11]. Microbiota maturation thus manifests as an increased ratio of anaerobic to facultative population levels, decreased population counts of different facultative bacteria and a paucity of *C. difficile* [8, 10, 11].

The most well studied factor disturbing gut microbiota development is delivery by cesarean section, which deprives the infant of contact with maternal fecal microbes such as *E. coli* and *Bacteroides*. Instead, cesarean-delivered infants acquire their bacteria from other sources, including *Enterobacteriaceae* species other than *E. coli* (e.g. *Klebsiella*, *Enterobacter*, etc.) and spore-forming clostridia that are present in the environment. Thus, infants delivered by cesarean section are colonized less often by *E. coli* and more often by other *Enterobacteriaceae* species than are vaginally delivered infants, and less often by *Bacteroides*, but more often by clostridia [8, 10, 12, 13]. In addition, antibiotics given during cesarean delivery may further impoverish the infant’s microbiota [14].

We were the first to note that the gut microbiota of firstborn infants appeared akin to that of cesarean-delivered infants, in that both groups had more clostridia and *Enterobacteriaceae* other than *E. coli* in their early microbiota [10], a finding confirmed by others [15]. We also noted a less prominent dominance of anaerobes in the gut microbiota of firstborn infants at one year of age, which we interpreted as a sign of an immature gut microbiota [10], as was supported by several later studies based on DNA sequencing [6, 9, 16–18]. However, the latter studies rarely permitted bacterial identification below the genus level and were not truly quantitative, but provided data on relative abundance of the different bacterial groups. Since several studies indicate that the very early gut microbiota may influence risk of allergy development [3–5], additional studies exploring gut microbiota development in the neonatal period in relation to birth order are needed.

Previously, we described the gut bacterial colonization pattern in the FARMFLORA birth cohort, using quantitative culture of major facultative and anaerobic bacteria from stool samples obtained regularly during infancy [19]. Here, the colonization pattern was related to parity (firstborn or having older siblings), delivery mode (cesarean section/vaginal delivery) and exposure to antibiotics during delivery, each adjusted for the other two exposures.

## Methods

### Study subjects

The current study analyzes the gut microbiota composition in the FARMFLORA birth cohort in relation to parity (firstborn or having older siblings), delivery mode (cesarean section/vaginal delivery) and exposure to antibiotics during delivery. Previously, we have published data from the same cohort, including the pattern of colonization of major facultative and anaerobic gut bacteria over the first year of life and the association between microbiota composition and farm *vs* non-farm residence, presence or absence of pets in the household, and to being diagnosed or not with allergy at 3 and 8 years of age, respectively [19]. A farming environment or pet exposure had modest effects on infant colonization pattern, while there was a strong link between infant microbiota colonization pattern and later allergy development, where e.g. a scarcity of bifidobacteria and a low anaerobe/facultative ratio in feces were risk factors for future allergy [19].

In brief, the FARMFLORA birth cohort comprises 65 infants born at term (≥ 38 gestational weeks) to families living in a rural area of southwest Sweden, around half of whom lived on small dairy farms [20]. Pregnant women were recruited in the period September 2005 – March 2008. On inclusion, women were interviewed by study nurses using questionnaires which included questions on e.g. family structure, parental allergy and pets in the household. Data on delivery mode, administration of antibiotics to the mother during delivery and time from rupture of membranes to delivery were retrieved by study nurses from hospital records (Supplemental Files S1-S2). Data on feeding pattern including breastfeeding and antibiotic treatments were recorded continuously by parents in journals (Supplemental Files S3-S4) and the information was collected when the child was 6, 12 and 18 months old [21]. Characteristics of the cohort are provided in Supplemental Table S1, and further details on the distribution of infants by year of birth are shown in Supplemental Table S2.

### Collection of samples and quantitative microbial culture

The data presented derive from a previous analysis of the gut bacterial colonization pattern in the FARMFLORA birth cohort, using quantitative culture of major facultative and anaerobic bacteria [19]. In brief, the gut microbiota was sampled on nine occasions from 3 days to 18 months of age. On day 3 after delivery, a rectal swab was collected by staff at the maternity ward, while freshly voided feces were collected by the parents at infants’ age 1, 2 and 4 weeks and 2, 4, 6, 12 and 18 months. The rectal swab was placed in transport medium, while the fecal samples were put in air-tight bags filled with an anaerobic atmosphere (AnaeroGen Compact, Oxoid Ltd, Basingstoke, UK) and transported to the laboratory where they were cultured within 24 h after collection. Culturing was carried out by the same experienced lab technicians throughout the study period.

Gut microbiota were analyzed by quantitative microbial culture focusing on bacterial groups known to dominate in the infant gut microbiota, i.e. *Bifidobacterium*, *Bacteroides*, *Lactobacillus*, *Clostridium*, *C. difficile* (aerotolerant and obligate anaerobes, termed “anaerobes”); *E. coli* and other *Enterobacteriaceae* (*Klebsiella*, *Enterobacter*, *Citrobacter*, *Proteus*, *Morganella*, *Raultella*, *Pantoea*, *Hafnia*), *Enterococcus*, *Staphylococcus aureus*, coagulase-negative staphylococci (CoNS), (facultatively anaerobic bacteria, termed “facultatives”).

The methodology used has been described in detail previously [19]. In brief, rectal swabs were streaked on non-selective and selective media that were cultured under aerobic conditions for identification of facultative bacteria (Supplemental Table S3) while fecal samples were diluted serially in sterile peptone water, cultured on a range of media and incubated both under aerobic and anaerobic conditions for isolation of facultative and anaerobic bacteria, respectively (Supplemental Table S3). Bacterial isolates were identified using biochemical and/or genetic methods (Supplemental Table S3) [22, 23], and their population counts were determined from agar plates of dilutions yielding 10–100 free-lying colonies. The limit of detection was 330 (10^2.52^) colony-forming units (CFU)/g feces.

For each sample, metrics for analysis were a) the presence/absence of each bacterial species/genus/group and b) its population count (CFU/g of feces; measured in colonized children only) and c) the ratio of population counts of anaerobic to facultative bacteria (anaerobic/facultative ratio), as a measure of the anaerobic character of the microbiota. The population count of facultatives was assessed by culture on non-selective medium under aerobic conditions. The population count of anaerobic bacteria was assessed by growth on non-selective medium under anaerobic conditions, from which we subtracted the counts of isolates which could also grow under aerobic conditions (determined by subculture).

### Statistical analyses

Participant characteristics were summarized using the median and interquartile range (IQR) for numeric variables, and counts and percentages for categorical variables. Baseline comparisons between exposure groups (birth order, delivery mode, and exposure to antibiotics during delivery) were performed using the Mann–Whitney U-test for numeric variables and Fisher’s exact test for binary variables.

Bacterial variables were presented as a) colonization rates, defined as the proportion (%) of infants harbouring the bacterium in question at each time-point; b) median population counts of each bacterium in colonized infants; and c) the median ratio of anaerobic to facultative bacterial counts at each time-point.

Gut microbiota composition was analyzed using generalized estimating equations (GEE) to account for intra-individual correlation in repeated measures. Continuous outcomes were modelled assuming a log-normal distribution, and binary variables using normal distribution with identity link. A Matern covariance matrix was applied to model temporal correlation, and robust standard errors (HC3 method) were used to account for violations against distributional assumptions. Time was treated as a categorical variable, including interactions with the exposure of interest to allow comparisons at each time point. Comparisons of colonization rates and population counts between groups at different time points were performed using Wald tests on the coefficients obtained from the GEE models.

Models were run both unadjusted and adjusted for the following potential confounders: birth order, delivery mode, exposure to antibiotics during delivery, breastfeeding (proportion of days of any breastfeeding from birth up to sampling) and infant sex. Results were reported as differences in colonization rates or as fold changes in population counts and anaerobic/facultative ratios.

All tests were two-sided and conducted at the 5% significance level. Statistical analyses were performed by using SAS/STAT® Software, version 9.4 (SAS Institute Inc. Cary, NC, USA) or IBM SPSS Statistics, version 29.0 (IBM Corp., Armonk, NY, USA). The dataset used in this study has been published separately in a data repository with restricted access [24].

## Results

**Fig. 1:**
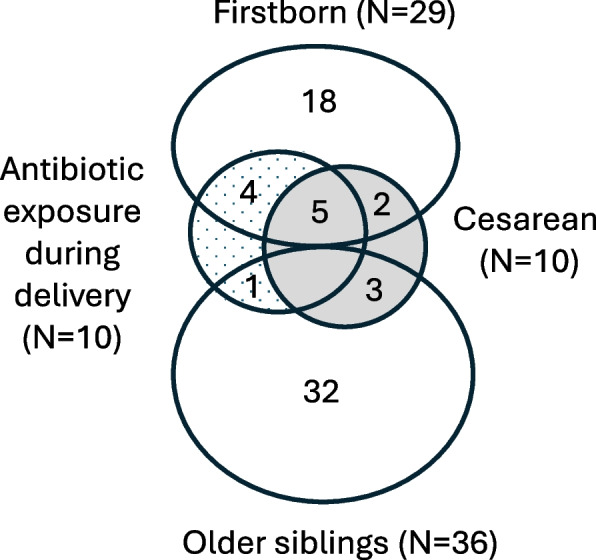
Venn diagram showing the number of infants who were firstborn (*N* = 29), delivered by cesarean section (*N* = 10), and/or exposed to antibiotics during delivery (*N* = 10)

Exposure group distributions are illustrated in Fig. 1. Among all participants, 29 (45%) were firstborn, 10 (15%) were delivered by cesarean section, and 10 (15%) were exposed to antibiotics during delivery. These three exposures were clearly interrelated. Firstborn infants were delivered by cesarean section three times as often as infants with older siblings (7/29 *vs* 3/36, *P* = 0.096), and were ten times more often exposed to antibiotics during delivery (9/29 *vs* 1/36, *P* = 0.004). Maternal antibiotics were administered in 50% of cesarean deliveries (5/10), compared to 9% of vaginal deliveries (5/55, *P* = 0.005).

### Colonization by major bacterial groups in firstborn infants compared to infants with older siblings

**Fig. 2:**
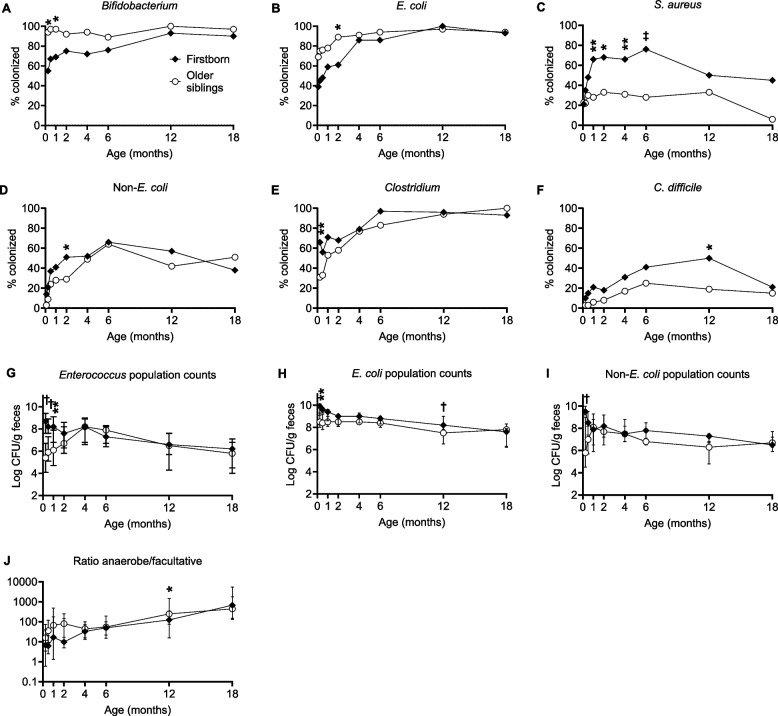
Colonization by major gut bacteria over the first 18 months in firstborn infants (*N* = 29) and in infants with older siblings (*N* = 36). **A–F** Proportion of infants (%) colonized by *Bifidobacterium*, *E. coli*, *S. aureus*, *Enterobacteriaceae* other than *E. coli* (non*-E. coli*), *Clostridium* spp. and *C. difficile*. **G–I** Fecal population counts (log CFU/g feces) of *Enterococcus, E. coli* and non*-E. coli* in colonized infants. **J** Ratio of anaerobic to facultative bacterial counts. Filled symbols represent the firstborn group; unfilled symbols represent infants with older siblings. For panels **G–J**, median values are presented and error bars denote the interquartile range. Asterisks denote statistically significant differences between groups after adjustment for delivery mode, antibiotic exposure during delivery, breastfeeding, and infant sex. *P < 0.05, **P < 0.01, †*P* < 0.001, Wald tests on coefficients obtained from Generalized Estimating Equations (GEE) models. The number of infants in each group colonized by each bacterium at each time point is shown in Supplemental Tables S4-S5

Figure 2 shows differences in colonization pattern between firstborn infants and infants with older siblings. The curves show unadjusted prevalence or population counts while asterisks denote significant differences between infant groups after adjustment for delivery mode, exposure to antibiotics during delivery, breastfeeding, and infant sex. Details of both unadjusted and adjusted associations are shown in Supplemental Tables S4 and S5, while major findings yielding significance in adjusted analyses are considered below.

Firstborn infants showed delayed acquisition of bifidobacteria (*P* = 0.031 and 0.018 at 1 week and 1 month respectively) and *E. coli* (*P* = 0.024 at 2 months) (Fig. 2A–B). Thus, while almost 100% of the infants with older siblings had acquired bifidobacteria by one week of age, this was true of only 55% of firstborn infants (Fig. 2A). *E. coli* was isolated from rectal swabs on day 3 in 69% of infants with older siblings, compared to a mere 39% of firstborn infants (Fig. 2B). Conversely, firstborn infants were more frequently colonized in the gut by *S. aureus* (*P* = 0.004, 0.016, 0.007 and < 0.0001 at 1, 2, 4 and 6 months respectively), by members of the *Enterobacteriaceae* family other than *E. coli* (denoted as “non-*E. coli*”, *P* = 0.040 at 2 months) and by anaerobic spore-formers, i.e. *Clostridium* spp. (*P* = 0.008 at 1 week) and *C. difficile* (*P* = 0.011 at 12 months) (Fig. 2C–F).

Among colonized children, firstborn infants had higher population counts of several facultative bacteria compared to infants with older siblings. This was most notable for *Enterococcus* spp. (*P* < 0.001, < 0.001 and 0.002 at 1 week, 2 weeks and 1 month respectively) (Fig. 2G), but also seen for *E. coli* (*P* = 0.009 and < 0.001 at 1 week and 12 months respectively) (Fig. 2H), and *Enterobacteriaceae* other than *E. coli* (*P* < 0.001 at 1 week) (Fig. 2I). Firstborn infants also had lower ratio of anaerobic to facultative bacterial counts in feces than infants with older siblings (*P* = 0.018 at 12 months) (Fig. 2J).

### Colonization by major bacterial groups in relation to delivery mode

**Fig. 3:**
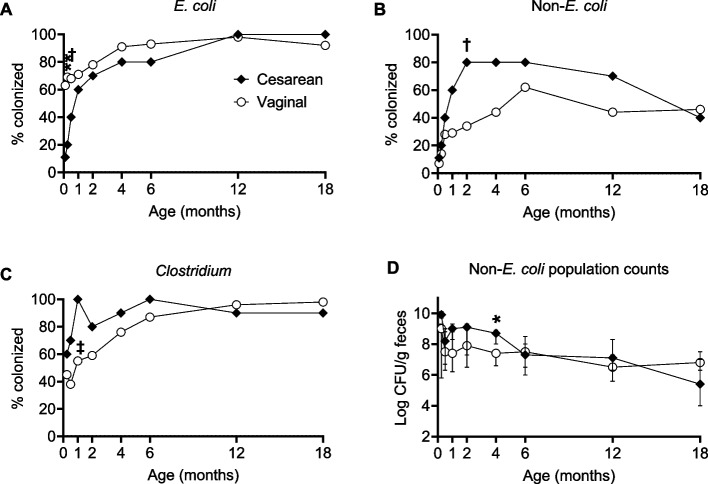
Colonization by major gut bacteria over the first 18 months in infants delivered by cesarean section (*N* = 10) or vaginally (*N* = 55). **A–C** Proportion of infants (%) colonized by *E. coli*, Enterobacteriaceae other than *E. coli* (non*-E. coli*) and *Clostridium* spp. **D** Fecal population counts (log CFU/g feces) of non*-E. coli* in colonized infants. Filled symbols represent the cesarean delivery group; unfilled symbols represent the vaginal delivery group. For panel **D**, median values are presented and error bars denote the interquartile range. Asterisks denote statistically significant differences between groups after adjustment for birth order, antibiotic exposure during delivery, breastfeeding, and infant sex. *P < 0.05, **P < 0.01, †*P* < 0.001, ‡*P* < 0.0001, Wald tests on coefficients obtained from Generalized Estimating Equations (GEE) models. The number of infants in each group colonized by each bacterium at each time point is shown in Supplemental Tables S6-S7

Figure 3 shows differences in colonization pattern between infants born by cesarean section and those born vaginally. Significant differences after adjustment for birth order, exposure to antibiotics during delivery, breastfeeding and infant sex are indicated by asterisks. Details of both unadjusted and adjusted associations are shown in Supplemental Tables S6 and S7, but only major findings that were significant in adjusted analyses are considered below.

In accordance with *E. coli* being confined to feces, to which cesarean-delivered infants are not exposed during delivery, there was a dramatic difference in colonization by *E. coli* between cesarean and vaginally-delivered infants on day 3 (11% vs. 63%, *P* = 0.002) and also at 1 week of age (20% vs. 69%, *P* < 0.001) (Fig. 3A). Conversely, cesarean-delivered infants were more frequently colonized by *Enterobacteriaceae* other than *E. coli* than vaginally delivered infants (*P* < 0.001 at 2 months) (Fig. 3B), and showed increased colonization by anaerobic spore-formers, i.e. *Clostridium* spp. (*P* < 0.0001 at 1 month) (Fig. 3C). Lastly, they also had higher population counts of *Enterobacteriaceae* other than *E. coli* when colonized by such bacteria (*P* = 0.010 at 4 months) (Fig. 3D).

### Colonization by major bacterial groups in relation to antibiotic treatment of the mother during delivery

**Fig. 4:**
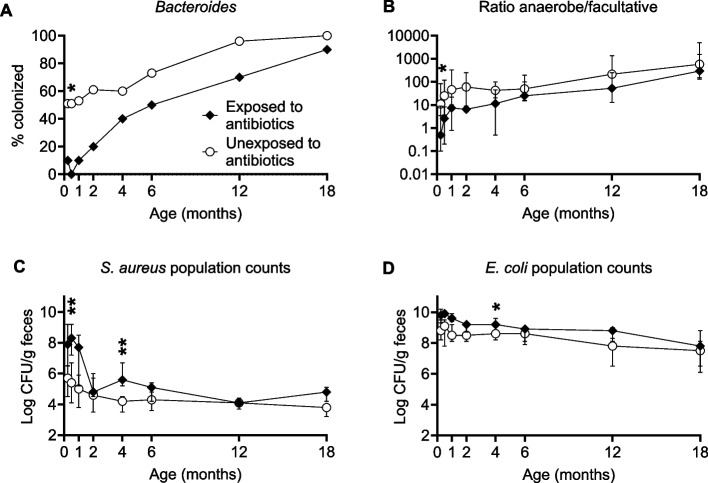
Colonization by major gut bacteria over the first 18 months in infants exposed (*N* = 10) or unexposed (*N* = 55) to antibiotics during delivery. **A:** Proportion of infants (%) colonized by *Bacteroides*. **B** Ratio of anaerobic to facultative bacterial counts. **C–D** Fecal population counts (log CFU/g feces) of *S. aureus* and *E. coli* in colonized infants. Filled symbols represent antibiotic-exposed infants; unfilled symbols represent unexposed infants. In panels **B–D,** median values are presented and error bars denote the interquartile range. Asterisks denote statistically significant differences between groups after adjustment for birth order, delivery mode, breastfeeding and infant sex. *P < 0.05, **P < 0.01, Wald tests on coefficients obtained from Generalized Estimating Equations (GEE) models. The number of infants in each group colonized by each bacterium at each time point is shown in Supplemental Tables S8-S9

Figure 4 shows the bacterial groups whose colonization was most clearly affected by administration of antibiotics to the mother during delivery; significances are calculated after adjustment for birth order, delivery mode, breastfeeding and sex. For full results, see Supplemental Table S8 and S9.

Infants exposed to antibiotics during delivery showed severely suppressed colonization by *Bacteroides* spp.. Only 10% acquired these bacteria in the first month, compared to 53% in unexposed infants (*P* = 0.046 at 2 weeks, Fig. 4A). They also had a lower ratio of anaerobic to facultative bacterial counts in the gut microbiota, which was significant at 1 week of age (*P* = 0.030) (Fig. 4B). When colonized by *S. aureus* during the first month, they had more than a 100-fold higher population counts of these bacteria compared to colonized unexposed infants (*P* = 0.007 at 2 weeks) (Fig. 4C); a less pronounced difference in *S. aureus* population counts was observed at 4 months (*P* = 0.004). The fecal population levels of *E. coli* were approximately tenfold lower in unexposed children at several timepoints (*P* = 0.010 at 4 months of age) (Fig. 4D).

### Summary of microbiota characteristics associated with being firstborn, delivered by caesarian section, and being exposed to maternal antibiotics during delivery

The main findings are summarized in Table 1, which shows the microbiota deviations associated with being firstborn, being delivered by cesarean section, or being exposed to maternal antibiotics during delivery, respectively. Significant deviations when compared to the corresponding reference groups are noted by arrows up or down, indicating increased or decreased colonization rate or population levels (in colonized infants) in the adjusted models.

**Table 1:** Gut microbiota characteristics significantly associated with birth order, delivery mode and exposure to antibiotics during delivery after adjustment for covariates

	Firstborn *vs* older sibling(s)		Cesarean *vs* vaginal delivery		Antibiotics during delivery: exposed vs unexposed	
**Colonization rate**						
Anaerobic bacteria						
* Bifidobacterium*	↓ *	1w, 1 m				
* Bacteroides*					↓ *	2w
* Clostridium*	↑ **	1w	↑ **‡**	1 m		
* C. difficile*	↑ *	12 m				
Facultative bacteria						
* E. coli*	↓ *	2 m	↓ **†**	3d, 1w		
Other *Enterobacteriaceae*	↑ *	2 m	↑ **†**	2 m		
* S. aureus*	↑ **	1–6 m				
**Population counts (colonized infants)**						
Facultative bacteria						
* E. coli*	↑ **†**	1w, 12 m			↑ *	4 m
Other *Enterobacteriaceae*	↑ **†**	1w	↑ *	4 m		
* Enterococcus*	↑ **†**	1–2w, 1 m				
* S. aureus*					↑ **	2w, 4 m
Anaerobe/facultative ratio	↓ *	12 m			↓ *	1w

Major findings from adjusted models are shown for each exposure. All models were adjusted for birth order, delivery mode, exposure to antibiotics during delivery, breastfeeding and infant sex. Only statistically significant associations (*P* < 0.05) are included. For full results, see Supplemental Tables S4–S9

*Abbreviations*: *d* day, *w* week, *m* month, ↑ higher colonization rate or population count in the exposed group, ↓ lower colonization rate or population count in the exposed group

^*^P < 0.05, **P < 0.01, **†***P* < 0.001, **‡***P* < 0.0001. For microbiota characteristics associated with an exposure at multiple sampling time points, the p-value of the strongest association is indicated

Overall, one may note that all the three investigated factors were, to a greater or lesser degree, associated with 1) less colonization by typical fecal bacteria (*Bifidobacterium*, *Bacteroides*, *E. coli*), 2) increased colonization by “opportunistic colonizers” easily picked up from sources other than the human gut (*S. aureus*, sporeforming clostridia and *C. difficile*, *Enterobacteriaceae* other than *E. coli*), 3) reduced anaerobe/facultative ratio in feces and 4) increased population levels of specific species or groups of facultative bacteria (Table 1).

Unique to being firstborn (after adjustment for the other factors), was a reduced colonization by *Bifidobacterium* spp. and an increased colonization by *S. aureus* and *C. difficile*, while reduced colonization by *Bacteroides* was a unique finding related to treatment with antibiotics during delivery.

## Discussion

In the present study, we analysed data from 65 infants followed from 3 days to 18 months of age with regular sampling of the fecal microbiota to determine whether being firstborn, delivered by cesarean section or indirectly exposed to antibiotics via the mother during delivery, were associated with deviations in gut microbiota development. These three “exposures” were highly interrelated – firstborn infants were more likely to have been delivered by cesarean section than infants with older siblings, while being firstborn or cesarean delivered were both associated with exposure to antibiotics during delivery. However, adjustment for these factors, and for breastfeeding and the infant’s sex, enabled us to assess the effects of these three exposures independent of one another.

In accordance with many previous studies [8, 25, 26], we demonstrated that cesarean delivered infants were colonized less often by *E. coli*, but more often by other *Enterobacteriaceae* and clostridia, than vaginally delivered infants. The latter bacterial groups may be regarded as “opportunistic” early colonizers, which readily establish in a poorly developed gut microbiota where competition is low [8]. We also noted delayed acquisition of *Bacteroides* in cesarean-delivered infants, in agreement with numerous studies [8], but significance was lost in the analysis adjusted for exposure to antibiotics during delivery; it is well known that acquisition of these bacteria is severely affected by intrapartum antibiotic exposure [14].

Interestingly, similar deviations as noted for cesarean-delivered infants characterized the microbiota of firstborn infants compared to those with older siblings; reduced acquisition of *E. coli* but increased colonization with other members of the *Enterobacteriaceae* family and with clostridial species. This largely agrees with our findings from a larger birth cohort, although we did not observe reduced colonization by *E. coli* in firstborn infants in this previous study [10]. Increased levels of clostridial species in firstborn infants have also been reported by others [15, 27].

A complex, mature microbiota suppresses the bacterial population numbers of facultative bacterial species [8, 28]. In line with firstborn infants having a less mature microbiota, they displayed considerably increased population counts of several facultative bacteria, including *E. coli*, other enterobacteria and enterococci. In fact, the enterococcal population levels were more than 100-fold higher in firstborn infants than in infants with older siblings during the first month of life. To the best of our knowledge, increased population counts of facultative bacteria in the first month of life in firstborn infants have not been reported previously.

The above observations indicate that firstborn infants are at least partially shielded from fecal bacteria in the first weeks of life. Although we cannot rule out older siblings as direct sources of fecal bacteria in the early neonatal period, it seems rather unlikely that they would transfer anaerobic bacteria in enough quantity and variety to strongly suppress facultative bacterial population sizes by at least two log units and already during the infant’s first week of life. We therefore hypothesize that transfer of fecal bacteria from the mother occurs less often during first deliveries than in subsequent ones. In subsequent deliveries, the pelvic floor may be weakened permitting a certain degree of fecal soiling to occur. Another possibility, not mutually exclusive, is that the more prolonged first delivery gives more time for enemas and other measures to prevent fecal contamination than subsequent deliveries that tend to occur more rapidly [29]. Accordingly, the time from rupture of membranes to delivery in the present cohort was longer in first deliveries as compared to second or third deliveries.

The firstborn infants in the present study showed reduced colonization by bifidobacteria in the first weeks of life, which is in line with our hypothesis of less transfer of fecal bacteria during first as compared to subsequent deliveries. However, bifidobacteria are probably quite easily acquired also from non-maternal sources, e.g. older siblings, since the majority of *sectio*-delivered infants catch up quite rapidly in terms of bifidobacterial colonization [8]. Other studies have reported lower amounts of bifidobacteria [17, 27, 30], or reduced prevalence of certain bifidobacterial species [31, 32], in the early gut microbiota of firstborn infants.

Interestingly, increased gut colonization by *S. aureus* in firstborn infants was one of the most marked findings in our study. Although *S. aureus* is generally thought of as a skin bacterium, it is a prominent member of the gut microbiota of Western infants, probably because their microbiota is less complex than that of infants living under less sanitary conditions [33, 34]. In firstborn infants, *S. aureus* was as common a colonizer as *E. coli* during the first two months, while infants with older siblings were at least twice as likely to carry *E. coli* than *S. aureus* in their gut microbiota during the same period. We have previously shown in another cohort that *S. aureus* strains colonizing the gut of infants often derive from the skin microbiota of the parents [33], but that many infants born to *S. aureus*-negative parents still become colonized by this bacterium, suggesting that other people may be the source. Firstborn infants may, for some reason, be handled by more different people than infants with older siblings, e.g. during a longer hospital stay. In addition, the less well developed gut microbiota of the firstborn infant may provide less resistance to the establishment and persistence of *S. aureus*. Hence, at 18 months of age, only two of the infants with older siblings had *S. aureus* in the gut microbiota, while this was true for 45% of the firstborn children (adj. *P* = 0.12). Interestingly, although antibiotic exposure was not associated with increased *S. aureus* colonization rate *per se*, *S. aureus* population levels in infants harbouring this bacterium were considerably higher if the infants had been exposed to antibiotics during partus, demonstrating the capacity of this opportunistic gut colonizer to expand in an immature gut microbiota depleted of anaerobes.

Finally, we also observed a lower ratio of anaerobes to facultatives at 12 months of age in firstborn infants, concomitant with an increased colonization by *C. difficile*, an anaerobic bacterium that is favoured in a poorly developed microbiota [11]**.** These findings are in agreement with earlier studies reporting delayed gut microbiota maturation in one year old firstborn infants [6, 10]. Other studies have also shown that firstborn infants have less of typical fecal anaerobes such as *Bacteroides* spp. [15] and *Faecalibacterium* [18] in their gut, further supporting poor microbiota development in these infants.

A limitation of the present study was the relatively small sample size, which reduces the statistical power to detect effects. Few infants in our cohort were delivered by cesarean section or exposed to antibiotics during delivery (*N* = 10 for each), which could potentially affect the reliability and generalizability of findings related to these factors. Also, due to the exploratory nature of this study we did not correct for multiple testing. However, several findings are consistent with those of previous studies. Bacterial culturing, as used in the current study, has both drawbacks and advantages. It misses most of the highly oxygen-sensitive anaerobes such as members of the *Ruminococcaceae* and *Lachnospiraceae* families that usually establish from around six months of age, unless multiple and advanced culture methods are used [35], and therefore does not generate a comprehensive description of the gut microbiota. However, in this study we concentrated on major groups of bacteria found in the early infant gut, such as bifidobacteria, lactobacilli, *Bacteroides,* members of *Clostridium sensu stricto* and *C. difficile*, as well as *E. coli, S. aureus* and enterococci, which are readily detected by culture. Furthermore, as a complex, mature microbiota rich in anaerobes suppresses the bacterial population numbers of facultative bacterial species [8, 28], the lack of a species-rich anaerobic microbiota in firstborn infants could be inferred from higher population counts of facultatives such as enterococci and *Enterobacteriaceae* in the first weeks after delivery. Another consequence of the fact that bacterial culturing misses highly oxygen-sensitive anaerobes is that the anaerobe/facultative ratio, which was determined here as a marker of gut microbiota maturity [8, 10], may be underestimated. However, an advantage of culturing was that it allowed us to quantify bacterial population counts, which otherwise requires a combination of DNA sequencing methods with either enumeration of bacterial cells or qPCR [36, 37].

A number of studies have identified firstborn children as more likely to develop allergy than those with older siblings [1, 2]. This may also apply, at least according to some studies, to children delivered by cesarean section. [38, 39] or exposed to antibiotics early in life [40]. In the present cohort, firstborn children were significantly more likely to have eczema at 8 years of age compared to children with older siblings (5/22 *vs.* 0/21, *P* = 0.048; unpublished data), and cesarean delivery as well as exposure to antibiotics during delivery were, or tended to be, associated with allergy at both 3 and 8 years of age [20, 41]. These associations may, at least partly, be mediated via altered intestinal colonization pattern, as we also observed significant links between allergy development and delayed acquisition of bifidobacteria and *Bacteroides*, low ratio of anaerobic to facultative bacterial population counts in feces and colonization by *C. difficile* [19]. Early bifidobacterial colonization was also associated with signs of both B- and T-cell maturation [42, 43]. The mechanism for the effect of the gut microbiota on immune development is unclear, but protection from eczema in children with older siblings in the present cohort was associated with higher fecal levels of the short-chain fatty acid valeric acid, which is produced by a complex anaerobic microbiota and has immune regulatory properties [44]. Other studies have also associated gut microbiota maturity at 1 year of age with protection from allergy [6] or asthma [7].

## Conclusions

Taken together, our results indicate that delivery by cesarean section, exposure to antibiotics during delivery and a lack of older siblings influence the gut colonization pattern in partly similar, partly unique ways that may, in turn, limit the ability of the commensal microbiota to educate the immune system during a critical window during infancy, which possibly results in poorly controlled immune responses and allergy development.

## Supplementary Information

Supplementary Material 1.

Supplementary Material 2.

## Data Availability

The data underlying the results of the present study have been deposited as a restricted data set in a data repository, the Swedish National Data Service (https://snd.se), with open metadata and documentation. It has been assigned a unique persistent identifier (DOI): 10.5878/ea19-6s81. To request access to the full dataset or parts of the dataset, a request management system is available in the repository for filing a formal inquiry to the University of Gothenburg. However, the data is subject to specific national confidentiality regulations (Public Access to Information and Secrecy Act, SFS 2009:400, https://www.riksdagen.se/sv/dokument-och-lagar/dokument/svensk-forfattningssamling/offentlighets-och-sekretesslag-2009400_sfs-2009–400/), and to the General Data Protection Regulation (GDPR), 2016/679, https://www.imy.se/en/organisations/data-protection/this-applies-accordning-to-gdpr/. This means that each request must be reviewed in a formal assessment by the University of Gothenburg, to examine if any data may be released in that specific case according to Swedish law.

## Abbreviations

CFU: Colony-forming units
IQR: Interquartile range
GEE: Generalized estimating equations
