# Supplementary material for: Major gut microbiota perturbations in firstborn infants compared to those with older siblings soon after delivery
Source: BMC Pediatr. 2025 Oct 7;25:780. doi: 10.1186/s12887-025-06015-7 (PMC12502136; doi:10.1186/s12887-025-06015-7)
Supplement: Supplementary file 1 — Supplementary Material 1. [file 12887_2025_6015_MOESM1_ESM.pdf]

Supplemental File S1. FARMFLORA Study Protocol - recruitment and data on pregnancy and delivery. Pages 1-9: Questionnaire delivered to participating women during interviews carried out on inclusion in the study. Pages 10-11: Form used to record pregnancy and delivery data retrieved from hospital records. (English translation of original Swedish language version)

Date filled in

YY	MM	DD

Date of birth

YY	MM	DD

4 last digits*

Child ID	

* Of Swedish personal number

Mother

Last name……………………………………..First name……………………………..

Swedish personal number……………………………………………………………….

Address……..…………………………………………………………………………...

………………………………………………….. Zip code ……………………..

Phone (home)………………………………Phone (work)……………………………

Phone (mobile)……………………………………………………………………

Father

Last name……………………………………..First name……………………………..

Swedish personal number……………………………………………………………….

Address……..…………………………………………………………………………...

………………………………………………….. Post code ……………………..

Phone (home)………………………………Phone (work)……………………………

Phone (mobile)……………………………………………………………………….

Siblings

First name	Sex	Year of birth

…………………………………………………………………………………………..

…………………………………………………………………………………………..

…………………………………………………………………………………………..

…………………………………………………………………………………………..

…………………………………………………………………………………………..

…………………………………………………………………………………………..

Mother		
**Mother**		
Mother's age		Years
Mother's occupation:		
Mother's education		
	Middle school (up to ~16 years)	
	High school (up to ~18 years)	
	Trade school *	
	College or university (≤ 3 years)	
	College or university (>3 years)	
* 'Gymnasieingenjör' or other trade-based training after high school		
	**No**	**Yes**
Has the mother ever had asthma?		
Has she had asthma symptoms in the last 12 months?		
Has she needed asthma treatment in the last 12 months?		
Has she ever had an asthma diagnosis (from a doctor)?		
Has the mother ever had allergic nasal or eye symptoms (allergic rhinoconjunctivitis (ARC))?		
Has she had ARC symptoms in the last 12 months?		
Has she needed ARC treatment in the last 12 months?		
Has she ever had an ARC diagnosis (from a doctor)?		
Has the mother ever had eczema?		
Has she had eczema symptoms in the last 12 months?		
Has she needed eczema treatment in the last 12 months?		
Has she ever had an eczema diagnosis (from a doctor)?		
Was the eczema contact dermatitis?		
Has the mother had any other allergic diseases?		
If so, which?		
Has she had symptoms in the last 12 months?		
Has she needed treatment in the last 12 months?		
Has this allergic disease been diagnosed by a doctor?		
Has the mother smoked in the last month?		
Did the mother smoke during gestational months 7-8?		
Did the mother smoke during gestational months 4-6?		
Did the mother smoke during gestational months 1-3?		
Did the mother use oral tobacco pouches ('snus') during pregnancy?		
Did the mother use nicotine gum or patches during pregnancy?		

Father			
**Father**			
Father’s age			Years
Father’s occupation:			
Father’s education			
	Middle school (up to ~16 years)		
	High school (up to ~18 years)		
	Trade school *		
	College or university (≤ 3 years)		
	College or university (>3 years)		
* ’Gymnasieingenjör’ or other trade-based training after high school			
		**No**	**Yes**
Has the father ever had asthma?			
Has he had asthma symptoms in the last 12 months?			
Has he needed asthma treatment in the last 12 months?			
Has he ever had an asthma diagnosis (from a doctor)?			
Has the father ever had allergic nasal or eye symptoms (allergic rhinoconjunctivitis (ARC))?			
Has he had ARC symptoms in the last 12 months?			
Has he needed ARC treatment in the last 12 months?			
Has he ever had an ARC diagnosis (from a doctor)?			
Has the father ever had eczema?			
Has he had eczema symptoms in the last 12 months?			
Has he needed eczema treatment in the last 12 months?			
Has he ever had an eczema diagnosis (from a doctor)?			
Was the eczema contact dermatitis?			
Has the father had any other allergic diseases?			
If so, which?			
Has he had symptoms in the last 12 months?			
Has he needed treatment in the last 12 months?			
Has this allergic disease been diagnosed by a doctor?			
Has the father smoked in the last month?			

Older sibling 1		
Sibling’s age	Years	
Sibling’s sex		
	**No**	**Yes**
Has the sibling ever had asthma?		
Has he/she had asthma symptoms in the last 12 months?		
Has he/she needed asthma treatment in the last 12 months?		
Has he/she ever had an asthma diagnosis (from a doctor)?		
Has the sibling ever had allergic nasal or eye symptoms (allergic rhinoconjunctivitis (ARC))?		
Has he/she had ARC symptoms in the last 12 months?		
Has he/she needed ARC treatment in the last 12 months?		
Has he/she ever had an ARC diagnosis (from a doctor)?		
Has the sibling ever had eczema?		
Has he/she had eczema symptoms in the last 12 months?		
Has he/she needed eczema treatment in the last 12 months?		
Has he/she ever had an eczema diagnosis (from a doctor)?		
Was the eczema contact dermatitis?		
Has the sibling had any other allergic diseases?		
If so, which?		
Has he/she had symptoms in the last 12 months?		
Has he/she needed treatment in the last 12 months?		
Has this allergic disease been diagnosed by a doctor?		

Older sibling 2		
Sibling’s age	Years	
Sibling’s sex		
	**No**	**Yes**
Has the sibling ever had asthma?		
Has he/she had asthma symptoms in the last 12 months?		
Has he/she needed asthma treatment in the last 12 months?		
Has he/she ever had an asthma diagnosis (from a doctor)?		
Has the sibling ever had allergic nasal or eye symptoms (allergic rhinoconjunctivitis (ARC))?		
Has he/she had ARC symptoms in the last 12 months?		
Has he/she needed ARC treatment in the last 12 months?		
Has he/she ever had an ARC diagnosis (from a doctor)?		
Has the sibling ever had eczema?		
Has he/she had eczema symptoms in the last 12 months?		
Has he/she needed eczema treatment in the last 12 months?		
Has he/she ever had an eczema diagnosis (from a doctor)?		
Was the eczema contact dermatitis?		
Has the sibling had any other allergic diseases?		
If so, which?		
Has he/she had symptoms in the last 12 months?		
Has he/she needed treatment in the last 12 months?		
Has this allergic disease been diagnosed by a doctor?		

Older sibling 3		
Sibling’s age	Years	
Sibling’s sex		
	**No**	**Yes**
Has the sibling ever had asthma?		
Has he/she had asthma symptoms in the last 12 months?		
Has he/she needed asthma treatment in the last 12 months?		
Has he/she ever had an asthma diagnosis (from a doctor)?		
Has the sibling ever had allergic nasal or eye symptoms (allergic rhinoconjunctivitis (ARC))?		
Has he/she had ARC symptoms in the last 12 months?		
Has he/she needed ARC treatment in the last 12 months?		
Has he/she ever had an ARC diagnosis (from a doctor)?		
Has the sibling ever had eczema?		
Has he/she had eczema symptoms in the last 12 months?		
Has he/she needed eczema treatment in the last 12 months?		
Has he/she ever had an eczema diagnosis (from a doctor)?		
Was the eczema contact dermatitis?		
Has the sibling had any other allergic diseases?		
If so, which?		
Has he/she had symptoms in the last 12 months?		
Has he/she needed treatment in the last 12 months?		
Has this allergic disease been diagnosed by a doctor?		

**Housing**			
Type of housing			
	Apartment		
	Terraced house		
	Detached house		
House/apartment area (in square meters)			
Total number of reception and bedrooms			
Type of ventilation			
	Natural ventilation		
	Mechanical ventilation		
	Does not know		
		**No**	**Yes**
Visible damp or mold, or >5cm height of condensation on double glazed windows			
House has a lower ground floor/furnished basement or is a slope house			
Number of adults living in the home			
Number of siblings (who primarily live with the child)			
Number of half siblings who live with the family at least 2 days out of every 2 weeks (at least every other weekend)			
Number of siblings who have had allergic diseases (asthma, ARC, eczema or food allergy)			
Number of siblings with asthma			
Number of siblings with ARC			
Number of siblings with eczema			
Number of siblings with food allergy			

Pet ownership		
Number of dogs in the home		
Number of cats in the home		
	**No**	**Yes**
Any pet rodents - indoors (rabbit, hamster etc…)		
Any pet rodents - outdoors (rabbit, hamster etc…)		
Any caged birds		
Any fish (aquarium)		
Any other animals – If yes, specify below		
Type of other animals		
If no dogs or cats: Have you avoided having dogs or cats because of allergy in the family (in the parents or siblings of the unborn child)		
	No, it was for other reasons	
	Yes, it was because of allergy in the family	

Pregnancy and delivery			
Duration of pregnancy		weeks	days
		No	Yes
Did the mother receive antibiotics during pregnancy?			
	Yes, give the number of courses		
	If yes, state if possible when, the specific antibiotic and the indication/reason		
		No	Yes
Did the mother receive antibiotics during delivery, or in the week after?			
	If yes, state if possible the specific antibiotic and the indication/reason		
Rupture of membranes			hours
		No	Yes
Antiseptic cleanse ('Hibiscrub') – shower			
Antiseptic cleanse ('Hibiscrub') – local			
Delivery mode (may select several options)			
	Vaginal		
	Lying down		
	Sitting		
	Birthing pool		
	Ventouse or forceps		
	Planned cesarean section		
	Acute cesarean section		
	Comments on delivery mode		

Child			
Sex	Boy		Girl
Birth weight			grams
Length			cm
Nutrition in maternity ward (may select several options)			
	Breast milk		
	Formula		
	Hydrolyzed formula (Nutramigen, Profylac, PeptiJunior)		
		**No**	**Yes**
Was the child considered healthy while in the maternity ward?			
	If no, which diseases/ health issues did the child have?		
	Congenital malformations/syndromes		
	Congenital heart defect		
	Light treatment for jaundice		
	Oral antibiotics		
	Intravenous antibiotics		
	Oxygen > 12 hours		
	CPAP		
	Respirator		
	Blood transfusion		
	Other health issues/conditions		
	Comments on health issues while in the maternity ward		
Number of days in the maternity ward			days
Number of days in the neonatal ward			days

Supplemental File S2. FARMFLORA Study Protocol - recruitment and data on pregnancy and delivery. Pages 1-9: Questionnaire delivered to participating women during interviews carried out on inclusion in the study. Pages 10-11: Form used to record pregnancy and delivery data retrieved from hospital records. (Original Swedish language version)

Ifyllt datum

YY	MM	DD

Födelsedatum

YY	MM	DD

4 sista siffror

Barn ID	
	sf+löpnr

Mamman

Efternamn………………………………………….. Förnamn……………………….

Personnr…………………………………………….

Adress……..…………………………………………………………………….

Postnr…………………………………………… Postadress……………………..

Tel hem………………………………………….. Tel arb…………………………

Mobiltel…………………………………………………………………………

Pappan

Efternamn………………………………………….. Förnamn……………………….

Personnr……………………………………………

Adress……..…………………………………………………………………….

Postnr…………………………………………… Postadress……………………..

Tel hem………………………………………….. Tel arb…………………………

Mobiltel…………………………………………………………………………

Syskon

Förnamn	kön	född år

…………………………………………………………………………………

………………………………………………………………………………….

………………………………………………………………………………….

………………………………………………………………………………….

………………………………………………………………………………….

………………………………………………………………………………….

Mamman		
**Mamman**		
Mammans ålder		År
Mammans yrke:		
Mammans utbildning		
	Grundskola	
	Gymnasium 2-3 år el motsvarande	
	Gymnasieingenjör eller annan eftergymnasial utb	
	Högskola eller universitet, max 120 poäng	
	Högskola el universitet > 120 poäng	

	**Nej**	**Ja**
Har mamman någonsin haft astma?		
Har hon haft astmabesvär sista 12 månaderna?		
Har hon behövt astmabehandling sista 12 månaderna		
Har hon någonsin fått diagnosen astma (av läkare)		
Har mamman någonsin haft allergisk snuva/ögonkatarr (ARC)?		
Har hon haft ARC-besvär sista 12 månaderna?		
Har hon behövt ARC-behandling sista 12 månaderna?		
Har hon någonsin fått diagnosen ARC (av läkare)?		
Har mamman någonsin haft eksem?		
Har hon haft eksembesvär sista 12 månaderna?		
Har hon behövt eksembehandling sista 12 månaderna?		
Har hon någonsin fått diagnosen eksem (av läkare)?		
Har eksemet varit av typ kontakteksem?		
Har mamman haft annan allergisk sjukdom?		
Om ja, vad?		
Har hon haft besvär av detta sista 12 månaderna?		
Har hon fått behandling för detta sista 12 månaderna?		
Har diagnosen ställts av läkare?		
Har mamman rökt sista månaden		
Rökte modern under graviditetsmånad 7 – 8		
Rökte modern under graviditetsmånad 4 – 6		
Rökte modern under graviditetsmånad 1 – 3		
Har modern snusat under graviditeten		
Har modern använt nikotintuggumi eller plåster under graviditeten		

Pappan			
**Pappan**			
Pappans ålder			År
Pappans yrke:			
Pappans utbildning			
	Grundskola		
	Gymnasium 2-3 år el motsvarande		
	Gymnasieingenjör eller annan eftergymnasial utb		
	Högskola eller universitet, max 120 poäng		
	Högskola el universitet > 120 poäng		
		**Nej**	**Ja**
Har pappan någonsin haft astma?			
Har han haft astmabesvär sista 12 månaderna?			
Har han behövt astmabehandling sista 12 månaderna			
Har han någonsin fått diagnosen astma (av läkare)			
Har pappan någonsin haft allergisk snuva/ögonkatarr (ARC)?			
Har han haft ARC-besvär sista 12 månaderna?			
Har han behövt ARC-behandling sista 12 månaderna?			
Har han någonsin fått diagnosen ARC (av läkare)?			
Har pappan någonsin haft eksem?			
Har han haft eksembesvär sista 12 månaderna?			
Har han behövt eksembehandling sista 12 månaderna?			
Har han någonsin fått diagnosen eksem (av läkare)?			
Har eksemet varit av typ kontakteksem?			
Har pappan haft annan allergisk sjukdom?			
Om ja, vad?			
Har han haft besvär av detta sista 12 månaderna?			
Har han fått behandling för detta sista 12 månaderna?			
Har diagnosen ställts av läkare?			
Har pappan rökt sista månaden			

Äldre syskon 1		
Syskonets ålder		År
Syskonets kön		
	**Nej**	**Ja**
Har syskonet någonsin haft astma?		
Har hon/han haft astmabesvär sista 12 månaderna?		
Har hon/han behövt astmabehandling sista 12 månaderna		
Har hon/han någonsin fått diagnosen astma (av läkare)		
Har syskonet någonsin haft allergisk snuva/ögonkatarr (ARC)?		
Har hon/han haft ARC-besvär sista 12 månaderna?		
Har hon/han behövt ARC-behandling sista 12 månaderna?		
Har hon/han någonsin fått diagnosen ARC (av läkare)?		
Har syskonet någonsin haft eksem?		
Har hon/han haft eksembesvär sista 12 månaderna?		
Har hon/han behövt eksembehandling sista 12 månaderna?		
Har hon/han någonsin fått diagnosen eksem (av läkare)?		
Har eksemet varit av typ kontakteksem?		
Har syskonet haft annan allergisk sjukdom?		
Om ja, vad?		
Har hon/han haft besvär av detta sista 12 månaderna?		
Har hon/han fått behandling för detta sista 12 månaderna?		
Har diagnosen ställts av läkare?		

**Äldre syskon 2**		
Syskonets ålder		År
Syskonets kön		
	**Nej**	**Ja**
Har syskonet någonsin haft astma?		
Har hon/han haft astmabesvär sista 12 månaderna?		
Har hon/han behövt astmabehandling sista 12 månaderna		
Har hon/han någonsin fått diagnosen astma (av läkare)		
Har syskonet någonsin haft allergisk snuva/ögonkatarr (ARC)?		
Har hon/han haft ARC-besvär sista 12 månaderna?		
Har hon/han behövt ARC-behandling sista 12 månaderna?		
Har hon/han någonsin fått diagnosen ARC (av läkare)?		
Har syskonet någonsin haft eksem?		
Har hon/han haft eksembesvär sista 12 månaderna?		
Har hon/han behövt eksembehandling sista 12 månaderna?		
Har hon/han någonsin fått diagnosen eksem (av läkare)?		
Har eksemet varit av typ kontakteksem?		
Har syskonet haft annan allergisk sjukdom?		
Om ja, vad?		
Har hon/han haft besvär av detta sista 12 månaderna?		
Har hon/han fått behandling för detta sista 12 månaderna?		
Har diagnosen ställts av läkare?		

**Äldre syskon 3**		
Syskonets ålder		År
Syskonets kön		
	**Nej**	**Ja**
Har syskonet någonsin haft astma?		
Har hon/han haft astmabesvär sista 12 månaderna?		
Har hon/han behövt astmabehandling sista 12 månaderna		
Har hon/han någonsin fått diagnosen astma (av läkare)		
Har syskonet någonsin haft allergisk snuva/ögonkatarr (ARC)?		
Har hon/han haft ARC-besvär sista 12 månaderna?		
Har hon/han behövt ARC-behandling sista 12 månaderna?		
Har hon/han någonsin fått diagnosen ARC (av läkare)?		
Har syskonet någonsin haft eksem?		
Har hon/han haft eksembesvär sista 12 månaderna?		
Har hon/han behövt eksembehandling sista 12 månaderna?		
Har hon/han någonsin fått diagnosen eksem (av läkare)?		
Har eksemet varit av typ kontakteksem?		
Har syskonet haft annan allergisk sjukdom?		
Om ja, vad?		
Har hon/han haft besvär av detta sista 12 månaderna?		
Har hon/han fått behandling för detta sista 12 månaderna?		
Har diagnosen ställts av läkare?		

**Bostad**			
Typ av bostad			
	Lägenhet		
	Radhus		
	Friliggande		
Bostadsyta i kvadratmeter			
Antal rum			
Typ av ventilation			
	Självdrag (köksfläkt kan ingå)		
	Mekanisk ventilation		
	Vet ej		
		**Nej**	**Ja**
Synlig fukt eller mögelskada, eller kondens vintertid > 5cm (tvåglas)			
Finns källare eller suterrängvåning			
Antal vuxna som bor i bostaden			
Antal syskon (som huvudsakligen bor med barnet)			
Antal ”halvsyskon” som bor med familjen minst 2 dagar var 14:e dag (minst var annan helg)			
Antal syskon som haft någon allergisk sjukdom (Astma, ARC, eksem eller födoämnesallergi)			
Antal syskon med astma			
Antal syskon med ARC			
Antal syskon med eksem			
Antal syskon med födoämnesallergi			

**Djurinnehav**		
Antal hundar i bostaden		
Antal katter i bostaden		
	**Nej**	**Ja**
Finns gnagare – inomhus (Kanin, hamster etc)		
Finns gnagare – utomhus (Kanin, hamster etc)		
Finns burfåglar		
Finns fiskar (akvarium)		
Finns annat djur – om ja, ange typ i kommentar nedan		
Kommentar till annat djur		
Om ej hund eller katt: Har ni avstått från att ha hund eller katt pga allergi i familjen (bland föräldrar och syskon till blivande barnet)		
	Nej, vi avstod av andra skäl	
	Ja, vi avstod pga allergi i familjen	

Graviditet och förlossning			
Graviditetslängd		vkr	dgr
		**Nej**	**Ja**
Antibiotika till modern under graviditeten?			
	Ja, ange antal kurer		
	Om ja, ange om möjligt när, preparat och indikation/orsak		
		**Nej**	**Ja**
Antibiotika till modern under förlossningen, och veckan efter?			
	Om ja, ange om möjligt preparat och indikation/orsak		
Vattenavgång			tim
		**Nej**	**Ja**
Hibiscrub – dusch			
Hibiscrub – lokalt			
Förlossningssätt (flera alternativ möjliga)			
	Vaginalt		
	Liggande		
	Sittande		
	Bubbelbad		
	Sugklocka eller tång		
	Planerat sectio		
	Akut sectio		
	Kommentar till förlossningssätt		

Barnet				
Kön	Pojke		Flicka	
Födelsevikt			gram	
Födelselängd			cm	
Tillmatning på BB (flera alternativ möjliga)				
	Bröstmjölk			
	Tillägg			
	Hydrolysat (Nutramigen, Profylac, PeptiJunior)			
			**Nej**	**Ja**
Har barnet uppfattats som friskt under BB-tiden				
	Om nej, vilka sjukdomar/problem har barnet haft?			
	Medfödd missbildning/syndrom			
	Hjärtfel			
	Ljusbehandling pga icterus			
	Antibiotika per os			
	Antibiotika i.v			
	Syrgas > 12 timmar			
	CPAP			
	Respirator			
	Blodtransfusion			
	Annat problem/sjd			
	Kommentar till sjukdom och problem under BB-tiden			
Antal dagar på BB			dgr	
Antal dagar på neonatalavdelning			dgr	

Supplemental File S3. FARMFLORA Study Journal. Provided to parents in order for them to continuously record information on breastfeeding, food introduction and illnesses. (English translation of original Swedish language version)

FARMFLORA Study Journal

This journal is intended to help you remember things we will ask you about at the planned interviews at 6 and 12 months of age, and at the doctor's examination at 18 months of age. Try to fill out the journal close in time to every important event, since it is easy to forget details otherwise.

We would like you to record how long the child was breastfed or received breastmilk, when different types of food were introduced and if the child was ill.

If the child was ill, we would like you to record the following information

Age (or date) when the child became ill

- What illness or symptoms the child had
- Number of days with fever > 38.5
- If the child had any breathing issues in connection with the illness (e.g. wheeze or labored breathing)
- Number of doses with anti-fever medications (paracetamol, ibuprofen)
- If the child received antibiotics, which type and for how many days?
- If the child had any other treatment (e.g. visits to the emergency department, admittance to hospital, inhalation treatment, cortisone tablets, i.v. fluids, surgery etc)
- Number of days the illness lasted

Diet

At what age (months and weeks) did you stop breastfeeding, or give the child formula, gruel ('välling') or a new food for the first time?

When did you stop breastfeeding or providing breastmilk?

……….………………………...

If the child was given any of the foods below (even pureed), when was it given for the first time?:

Formula ……………………………………

Gruel (gluten free) ……………………………………

Gruel (with gluten) ……………………………………

Potatoes or root vegetables……………………………………

Vegetables ……………………………………

Soy, peas, beans, lentils ……………………………………

Fruit ……………………………………

Berries ……………………………………

Peanuts …………………………………

Hazelnuts/almonds …………………………………
(including marzipan and hazelnut paste in baked goods)

Meat (any kind) …………………………………

Egg …………………………………
(including in food and baked goods)

Fish …………………………………

Bread, wheat crackers, pasta…………………………………

Dairy products …………………………………
(milk, butter, margarine, yoghurt, vanilla ice cream etc)

Illnesses

Age or date ……………………………………………………………..

Diagnosis or symptoms ……………………………………………

………………………………………………………………………

Number of days with fever …………………………………………
(over 38.5)

Breathing issues (if any) ……………………………………………
(Wheeze or labored breathing)

Anti-fever medications ……………………………………………
(type and number of doses)

Antibiotics ……………………………………………
(type and number of doses)

Other treatment and visits to emergency department, admittance to hospital etc (if any)
………………………………………………………………………

………………………………………………………………………

………………………………………………………………………

Number of days the illness lasted……………………………………

Illnesses

Age or date ……………………………………………………………..

Diagnosis or symptoms ……………………………………………

………………………………………………………………………

Number of days with fever …………………………………………
(over 38.5)

Breathing issues (if any) ……………………………………………
(Wheeze or labored breathing)

Anti-fever medications ……………………………………………
(type and number of doses)

Antibiotics ……………………………………………
(type and number of doses)

Other treatment and visits to emergency department, admittance to hospital etc (if any)
………………………………………………………………………

………………………………………………………………………

………………………………………………………………………

Number of days the illness lasted……………………………………

Illnesses

Age or date ……………………………………………………………..

Diagnosis or symptoms ……………………………………………

………………………………………………………………………

Number of days with fever …………………………………………
(over 38.5)

Breathing issues (if any) ……………………………………………
(Wheeze or labored breathing)

Anti-fever medications ……………………………………………
(type and number of doses)

Antibiotics ……………………………………………
(type and number of doses)

Other treatment and visits to emergency department, admittance to hospital etc (if any)
………………………………………………………………………

………………………………………………………………………

………………………………………………………………………

Number of days the illness lasted……………………………………

Illnesses

Age or date ……………………………………………………………..

Diagnosis or symptoms ……………………………………………

………………………………………………………………………

Number of days with fever …………………………………………
(over 38.5)

Breathing issues (if any) ……………………………………………
(Wheeze or labored breathing)

Anti-fever medications ……………………………………………
(type and number of doses)

Antibiotics ……………………………………………
(type and number of doses)

Other treatment and visits to emergency department, admittance to hospital etc (if any)
………………………………………………………………………

………………………………………………………………………

………………………………………………………………………

Number of days the illness lasted……………………………………

Illnesses

Age or date ……………………………………………………..

Diagnosis or symptoms ………………………………………

………………………………………………………………

Number of days with fever ……………………………………… (over 38.5)

Breathing issues (if any) ……………………………………… (Wheeze or labored breathing)

Anti-fever medications ……………………………………… (type and number of doses)

Antibiotics ……………………………………… (type and number of doses)

Other treatment and visits to emergency department, admittance to hospital etc (if any)

………………………………………………………………

………………………………………………………………

………………………………………………………………

Number of days the illness lasted…………………………………

Phone numbers and staff contacts in the FARMFLORA study

XX	Study coordinator	XXX
XX	Study coordinator	XXX
XX	Pediatric nurse, Mariestad	XXX
XX	Pediatric nurse, Vara	XXX
XX	Pediatrician, Falköping	XXX
XX	Pediatrician, Lidköping	XXX
XX	Pediatrician, Vara	XXX
XX	Pediatrician, Skövde	XXX
XX	Pediatrician, Gbg	XXX
XX	Bacteriologist	XXX
XX	Bacteriologist	XXX
XX	Laboratory assistants	XXX
XX		
XX		
XX	Nutritionist researcher	XXX
XX	Site manager, Swedish university of agricultural sciences, SLU, Skara	XXX

Supplemental File S4. FARMFLORA Study Journal. Provided to parents in order for them to continuously record information on breastfeeding, food introduction and illnesses. (Original Swedish language version)

Dagbok i bondgårds-FLORA-studien

Dagboken är till för att hjälpa er komma ihåg sådant vi kommer att fråga om vid de planerade intervjuerna som sker vid 6 och 12 månaders ålder, och vid läkarundersökningen vid 18 månaders ålder. Försök fylla i dagboken i nära anslutning till varje viktig händelse, eftersom det är lätt att glömma alla detaljer.

Det vi vill att ni skall anteckna är hur länge barnet ammades eller fick bröstmjölk, när olika sorters mat introducerades samt om barnet varit sjukt.

Vid sjukdom är det nedanstående uppgifter vi vill att ni skriver ner

- Ålder (eller datum) när barnet blev sjukt
- Vad barnet hade för sjukdom eller symtom
- Antal dagar med feber över 38.5
- Om barnet haft andningsbesvär i samband med infektionen (t ex pip i bröstet eller tung, ansträngd andning)
- Antal doser med febernedsättande (Panodil, Alvedon, Brufen eller Ipren, Ibumetin)
- Om barnet fått antibiotika, vilken typ och hur många dagar
- Om barnet fått någon annan behandling (t ex akutbesök, inläggning, inhalationsbehandling, kortisontableter, dropp, operation etc)
- Antal dagar som sjukdomen varade

Uppfödning

Ange barnets ålder i månader och veckor när ni slutade amma eller första gången gav tillägg/välling eller ny mat

När slutade ni amma, ge bröstmjölk…………….…………….

Om barnet fått något av nedanstående (även som pure), när gavs det första gången:

Tillägg ……………………………………

Välling (glutenfri) ……………………………………

Välling (med gluten) ……………………………………

Potatis o rotfrukter ……………………………………

Grönsaker ……………………………………

Soja, ärtor, bönor, linser ……………………………………

Frukt ……………………………………

Bär ……………………………………

Jordnötter ……………………………………

Hasselnöt/Mandel ……………………………………
(även mandelmassa och hasselnötskräm i bakverk)

Kött (alla sorter) …………………………………

Ägg …………………………………
(även ägg i mat och bakverk)

Fisk …………………………………

Bröd, rån, pasta …………………………………

Mjölkprodukter …………………………………
(mjölk, smör, margarin, bordsmargarin, yoghurt, vaniljglass etc)

Sjukdomsepisoder

Ålder eller datum ……………………………………….

Diagnos eller symtom ……………………………………….

…………………………………………………………………….

Antal dagar med feber ……………………………………….
(över 38.5)

Ev andningsbesvär ……………………………………….
(pip i brötet eller ansträngd andning)

Febernedsättande ……………………………………….
(sort och antal doser)

Antibiotika ……………………………………….
(sort och antal dagar)

Annan behandling och ev akutbesök, sjukhusinläggning etc

…………………………………………………………………….

…………………………………………………………………….

…………………………………………………………………….

Antal dagar sjukdomen varade …………………………..

Sjukdomsepisoder

Ålder eller datum ……………………………………….

Diagnos eller symtom ……………………………………….

…………………………………………………………………….

Antal dagar med feber ……………………………………….
(över 38.5)

Ev andningsbesvär ……………………………………….
(pip i brötet eller ansträngd andning)

Febernedsättande ……………………………………….
(sort och antal doser)

Antibiotika ……………………………………….
(sort och antal dagar)

Annan behandling och ev akutbesök, sjukhusinläggning etc

…………………………………………………………………….

…………………………………………………………………….

…………………………………………………………………….

Antal dagar sjukdomen varade …………………………..

Sjukdomsepisoder

Ålder eller datum ………………………………………….

Diagnos eller symtom ………………………………………….

…………………………………………………………………………

Antal dagar med feber ………………………………………….
(över 38.5)

Ev andningsbesvär ………………………………………….
(pip i brötet eller ansträngd andning)

Febernedsättande ………………………………………….
(sort och antal doser)

Antibiotika ………………………………………….
(sort och antal dagar)

Annan behandling och ev akutbesök, sjukhusinläggning etc

…………………………………………………………………………

…………………………………………………………………………

…………………………………………………………………………

Antal dagar sjukdomen varade …………………………..

Sjukdomsepisoder

Ålder eller datum ………………………………………….

Diagnos eller symtom ………………………………………….

…………………………………………………………………………

Antal dagar med feber ………………………………………….
(över 38.5)

Ev andningsbesvär ………………………………………….
(pip i brötet eller ansträngd andning)

Febernedsättande ………………………………………….
(sort och antal doser)

Antibiotika ………………………………………….
(sort och antal dagar)

Annan behandling och ev akutbesök, sjukhusinläggning etc

…………………………………………………………………………

…………………………………………………………………………

…………………………………………………………………………

Antal dagar sjukdomen varade …………………………..

Sjukdomsepisoder

Ålder eller datum ………………………………………….

Diagnos eller symtom ………………………………………….

……………………………………………………………………….

Antal dagar med feber ………………………………………….
(över 38.5)

Ev andningsbesvär ………………………………………….
(pip i brötet eller ansträngd andning)

Febernedsättande ………………………………………….
(sort och antal doser)

Antibiotika ………………………………………….
(sort och antal dagar)

Annan behandling och ev akutbesök, sjukhusinläggning etc

……………………………………………………………………….

……………………………………………………………………….

……………………………………………………………………….

Antal dagar sjukdomen varade …………………………..

Telefonnr och kontaktpersoner i bondgårds-FLORA-studien

XX	Studiekoordinator	XXXX
XX	Studiekoordinator	XXXX
XX	Barnsjuksköt, Mariestad	XXXX
XX	Barnsjuksköt, Vara	XXXX
XX	Barnläkare, Falköping	XXXX
XX	Barnläkare, Lidköping	XXXX
XX	Barnläkare, Vara	XXXX
XX	Barnläkare, Skövde	XXXX
XX	Barnläkare, Gbg	XXXX
XX	Bakteriolog	XXXX
XX	Bakteriolog	XXXX
XX	Laboratorieassistenter	XXXX
XX		
XX		
XX	Näringsforskare	XXXX
XX	Platschef, SLU, Skara	XXXX
