# Supplementary material for: Major gut microbiota perturbations in firstborn infants compared to those with older siblings soon after delivery
Source: BMC Pediatr. 2025 Oct 7;25:780. doi: 10.1186/s12887-025-06015-7 (PMC12502136; doi:10.1186/s12887-025-06015-7)
Supplement: Supplementary file 2 — Supplementary Material 2. [file 12887_2025_6015_MOESM2_ESM.pdf]

Supplemental Table S1. Characteristics of the FARMFLORA cohort and comparisons between firstborn infants and infants with older siblings, infants delivered by cesarean section and vaginally, and infants exposed or not exposed to antibiotics during delivery.

		Firstborn		Cesarean delivery		Antibiotics during delivery	
**Child characteristics**	**Total cohort (N=65)**	**Yes (N=29)**	**No (N=36)**	**Yes (N=10)**	**No (N=55)**	**Yes (N=10)**	**No (N=55)**
First-born[a]	29 (45)	-	-	7 (70)	22 (40)	9 (90)	20 (36) **
Cesarean delivery[a]	10 (15)	7 (24)	3 (8.3)	-	-	5 (50)	5 (9.1) **
Antibiotics during delivery[a,b]	10 (15)	9 (31)	1 (3) **	5 (50)	5 (9.1) **	-	-
Time from rupture of membranes to vaginal delivery[c]	4.5 (1–15)	10 (3–37)	3.0 (1–9) **	-	4.5 (1–15)	39 (5–57)	4.0 (1–12)
Girl	32 (49)	14 (48)	18 (50)	4 (40)	28 (51)	3 (30)	29 (53)
Farming family	28 (43)	10 (35)	18 (50)	3 (30)	25 (46)	2 (20)	26 (47)
Pets in household[d]	40 (63)	19 (68)	21 (58)	7 (70)	33 (61)	6 (60)	34 (63)
Exclusively breastfed at 4 mo[e]	34 (52)	15 (52)	19 (53)	3 (30)	31 (56)	5 (50)	29 (53)
Partially breastfed at 12 mo[e]	33 (51)	15 (52)	18 (50)	3 (30)	30 (55)	4 (40)	29 (53)
Antibiotics at 0–6 months[f]	11 (18)	5 (19)	6 (17)	2 (20)	9 (17)	3 (33)	8 (15)
Antibiotics at 6–12 months[f]	11 (17)	4 (14)	7 (19)	4 (40)	7 (13)	3 (30)	8 (15)
Antibiotics at 12–18 months[f]	17 (27)	8 (28)	9 (27)	6 (60)	11 (21) *	5 (50)	12 (23)

Data are presented as numbers (percentages) of infants, or median (interquartile range). Comparisons between groups were performed using Fisher's Exact test or Mann-Whitney U-Test, and significant associations are indicated by * $p < 0.05$, ** $p < 0.01$. [a] See **Supplemental Table S2** for distribution of infants by year of birth. [b] Data on maternal antibiotic treatment during delivery were retrieved from hospital records. Women were treated with erythromycin (N=2), cefuroxime (N=2), cefuroxime and metronidazole (N=2), benzylpenicillin (N=1) and benzylpenicillin and cefuroxime (N=1); data on the type of antibiotic was missing for 2 treated women. [c] Data were retrieved from hospital records and were available for 48 vaginal deliveries; time in hours. [d] Data regarding pets (cat and/or dog) in household was missing for one child. [e] Parents recorded details of breastfeeding continuously in diaries, which were collected by study personnel when the child was 6, 12 and 18 months old (Jonsson, K. et al. Late Introduction of Fish and Eggs Is Associated with Increased Risk of Allergy Development - Results from the Farmflora Birth Cohort. *Food Nutr Res* **61**, 1393306 (2017)). Partial breastfeeding refers to any breastfeeding as part of infants´ diet. [f] Antibiotics refer to antibiotics given to infants. Data on the specific antibiotics, and the numbers of children treated with each, have been described previously (S1 Table in Ljung, A. et al. Gut Microbiota Markers in Early Childhood Are Linked to Farm Living, Pets in Household and Allergy. *PLoS One* **19**, e0313078 (2024)).

Supplemental Table S2. Distribution of firstborn infants, infants delivered by cesarean section, infants exposed to antibiotics during delivery, and infants from farming families, by year of birth

	2005	2006	2007	2008	p-value[a]
First-born	3/3 (100)	13/36 (36)	11/22 (50)	2/4 (50)	1.00
Cesarean delivery	0/3 (0)	8/36 (22)	2/22 (9)	0/4 (0)	0.32
Antibiotics during delivery	0/3 (0)	8/36 (22)	2/22 (9)	0/4 (0)	0.32
Farming family	1/3 (33)	13/36 (36)	12/22 (55)	2/4 (50)	0.27

Data are presented as numbers (percentages) of infants. [a] Distribution of infants in each group by year of birth was assessed using Linear-by-Linear association.

Supplemental Table S3. Culture media, growth conditions and identification methods used for the isolation, identification and enumeration of bacteria that are commonly present in the early gut microbiota.

Bacteria	Culture medium	Incubation conditions	Time (days)	Method of bacterial identification
Total facultatives	Colombia blood agar [1]	Air	2	Growth under aerobic conditions
Enterobacteriaceae	Drigalski agar [2]	Air	2	Gram-stain, API 20E biotyping system (Bio-Merieux, Marcy l'Etoile, France)
Staphylococcus	Staphylococcus agar [3]	Air	2	Gram-stain, Coagulase test
Enterococcus	Enterococcosel agar [4]	Air	2	Gram-stain, Esculine test
Total anaerobes[a]	Brucella blood agar [5]	Anaerobic	3	Growth under anaerobic but not aerobic conditions[a]
Bacteroides	Bacteroides bile esculine agar [6]	Anaerobic	3	Gram-stain, Rapid ID 32 A biotyping system (Bio-Merieux).
Bifidobacterium	Beerens agar [7]	Anaerobic	3	Gram-stain, Genus-specific Real-Time PCR assay[c]
Lactobacillus	Rogosa agar [8]	Anaerobic	3	Gram-stain, Multiplex group-specific PCR assay[d]
Clostridium[b]	Brucella blood agar [5]	Anaerobic	3	Gram-stain[e], Rapid ID 32 A biotyping system (Bio-Merieux).
Clostridioides difficile	CCFA agar [9]	Anaerobic	3	Gram-stain, Rapid ID 32 A biotyping system (Bio-Merieux).

Rectal swabs (aerobic culture only) and serially diluted fecal samples (quantitative aerobic and anaerobic cultures) were incubated at 37°C on the above-listed media for the isolation and enumeration of the indicated bacterial groups. The methods used for culturing and identification have previously been described in detail (S1 Appendix in [10]). The total facultative bacterial count of a sample was determined from the bacterial population counts on Colombia blood agar incubated in air, while the total anaerobic bacterial count was determined from the anaerobic bacterial population counts on Brucella blood agar incubated anaerobically. [a] Isolates growing both aerobically and anaerobically when subcultured were not included in the total anaerobic bacterial counts, with the exception of Gram-positive rods that exhibited weak growth under aerobic conditions (typically *Lactobacillus* or *Bifidobacterium* spp.). [b] For the enumeration of *Clostridium* spp. (anaerobic spore-formers, including also *C. difficile*), fecal samples diluted 1:10 were mixed with 99% ethanol and incubated on a shaker at room temperature for 30 min, to kill vegetative cells. Thereafter, the samples were serially diluted and plated on Brucella blood agar. [c] *Bifidobacterium* spp. were identified in a genus-specific Real-Time PCR assay [11]. Some isolates that failed to grow on subculturing were defined as bifidobacteria based on their Gram-stained appearance (i.e., bifid or club-shaped bacterial forms). [d] *Lactobacillus* spp. were identified using a *Lactobacillus* group-specific multiplex PCR assay [12,13]. [e] Some spore-forming Gram-positive or Gram-variable rod-shaped anaerobic bacteria failed to grow on subculturing but were accepted as *Clostridium* spp. based on appearance in the Gram staining.

References for Supplemental Table S3:

1 Ellner, P. D., Stoessel, C. J., Drakeford, E. & Vasi, F. A New Culture Medium for Medical Bacteriology. *Am J Clin Pathol* **45**, 502-504 (1966).

2 Kauffman, F. *The Bacteriology of Enterobacteriaceae 2nd Ed.* 362–363 (Munkgaard, 1969).

3 Chapman, G. H. Comparison of Ludlam's Medium with Staphylococcus Medium Number 110 for the Isolation of Staphylococci That Clot Blood. *J Bacteriol* **58**, 823 (1949).

4 Isenberg, H. D., Goldberg, D. & Sampson, J. Laboratory Studies with a Selective Enterococcus Medium. *Appl Microbiol* **20**, 433-436 (1970).

5 Slots, J. Bacterial Specificity in Adult Periodontitis. A Summary of Recent Work. *J Clin Periodontol* **13**, 912-917 (1986).

6 Livingston, S. J., Kominos, S. D. & Yee, R. B. New Medium for Selection and Presumptive Identification of the Bacteroides Fragilis Group. *J Clin Microbiol* **7**, 448-453 (1978).

7 Silvi, S., Rumney, C. J. & Rowland, I. R. An Assessment of Three Selective Media for Bifidobacteria in Faeces. *J Appl Bacteriol* **81**, 561-564 (1996).

8 Rogosa, M., Mitchell, J. A. & Wiseman, R. F. A Selective Medium for the Isolation and Enumeration of Oral and Fecal Lactobacilli. *J Bacteriol* **62**, 132-133 (1951).

9 George, W. L., Sutter, V. L., Citron, D. & Finegold, S. M. Selective and Differential Medium for Isolation of Clostridium Difficile. *J Clin Microbiol* **9**, 214-219 (1979).

10 Ljung, A. et al. Gut Microbiota Markers in Early Childhood Are Linked to Farm Living, Pets in Household and Allergy. *PLoS One* **19**, e0313078 (2024).

11 Penders, J. et al. Quantification of Bifidobacterium Spp., Escherichia Coli and Clostridium Difficile in Faecal Samples of Breast-Fed and Formula-Fed Infants by Real-Time Pcr. *FEMS Microbiol Lett* **243**, 141-147 (2005).

12 Ahrne, S. et al. Lactobacilli in the Intestinal Microbiota of Swedish Infants. *Microbes Infect* **7**, 1256-1262 (2005).

13 Song, Y. et al. Rapid Identification of 11 Human Intestinal Lactobacillus Species by Multiplex Pcr Assays Using Group- and Species-Specific Primers Derived from the 16s-23s Rrna Intergenic Spacer Region and Its Flanking 23s Rrna. *FEMS Microbiol Lett* **187**, 167-173 (2000).

Supplemental Table S4. Bacterial variables with colonization rate associated with birth order, unadjusted and adjusted for delivery mode, exposure to antibiotics during delivery, breastfeeding and infant sex.

		**Percent difference in colonization rate (95% CI) Firstborn children vs children with older siblings**			
**Variable and age at sampling**	**n (%) colonized in firstborn / older siblings**	**Unadjusted**	**Adjusted**	**Higher (↑) / Lower (↓) in firstborn group**	
Bifidobacterium colonization at 1 w	16 (55) / 30 (94)	-39 (-59 to -18) †	-27 (-51 to -2) *		↓
Bifidobacterium colonization at 2 w	18 (67) / 32 (97)	-28 (-47 to -9) **	-20 (-42 to +1)		↓
Bifidobacterium colonization at 1 mo	20 (69) / 35 (97)	-28 (-47 to -10) **	-26 (48 to -5) *		↓
Bifidobacterium colonization at 4 mo	21 (72) / 33 (94)	-21 (-40 to -2) *	-15 (-36 to +6)		↓
E. coli colonization at 3 d	11 (39) / 24 (69)	-26 (-51 to -2) *	-16 (-44 to +12)		↓
E. coli colonization at 1w	13 (45) / 24 (75)	-27 (-51 to -3) *	-20 (-48 to -9)		↓
E. coli colonization at 2 w	13 (48) / 25 (76)	-25 (-49 to -2) *	-21 (-50 to +9)		↓
E. coli colonization at 2 mo	17 (61) / 32 (89)	-27 (-49 to -6) *	-29 (-55 to -4) *		↓
S. aureus colonization at 1 mo	19 (66) / 10 (28)	+38 (+14 to +61) **	+39 (+12 to +65) **	↑	
S. aureus colonization at 2 mo	19 (68) / 12 (33)	+33 (+9 to +57) **	+34 (+6 to +62) *	↑	
S. aureus colonization at 4 mo	19 (66) / 11 (31)	+35 (+11 to +59) **	+39 (+11 to +68) **	↑	
S. aureus colonization at 6 mo	22 (76) / 10 (28)	+48 (+26 to +70) ‡	+57 (+32 to +83) ‡	↑	
S. aureus colonization at18 mo	13 (45) / 2 (6)	+38 (+18 to +59) †	+35 (-10 to +80)	↑	
Non-*E. coli* colonization at 2 mo	16 (57) / 10 (29)	+29 (+4 to +53) *	+28 (+1 to +54) *	↑	
Clostridium colonization at 1w	19 (66) / 10 (31)	+34 (+10 to +59) **	+37 (+10 to +65) **	↑	
C. difficile colonization at 12 mo	14 (50) / 7 (19)	+31 (+8 to +55) **	+49 (+11 to +87) *	↑	

Statistical analyses were performed using generalized estimating equations (GEE) to account for intra-individual correlation in repeated measures. Results are presented as difference in colonization rate of bacterial variables associated with birth order, unadjusted and adjusted for potential confounders, with 95% confidence intervals (CIs). Significant differences between groups are indicated by * $p < 0.05$, ** $p < 0.01$, † $p < 0.001$, ‡ $p < 0.0001$, Wald tests on coefficients obtained from GEE models.

Supplemental Table S5. Bacterial variables with population counts in colonized children associated with birth order, unadjusted and adjusted for delivery mode, exposure to antibiotics during delivery, breastfeeding and infant sex.

		**Fold change in population counts in colonized children (Fold-change (95% CI)) Firstborn children vs children with older siblings**			
**Variable and age at sampling**	**n (%) colonized in firstborn / elder siblings**	**Unadjusted**	**Adjusted**	**Higher (↑) / Lower (↓) in firstborn group**	
Ratio anaerobe/facultative at 1 w	-	0.20 (0.05 to 0.86) *	0.33 (0.07 to 1.6)		↓
Ratio anaerobe/facultative at 12 mo	-	0.27 (0.09 to 0.83) *	0.14 (0.03 to 0.71) *		↓
E. coli counts at 1 w	13 (45) / 24 (75)	11 (2.5 to 52) **	11 (1.9 to 67) **	↑	
E. coli counts at 2 w	13 (48) / 25 (76)	6.9 (1.3 to 37) *	4.5 (0.63 to 32)	↑	
E. coli counts at 1 mo	17 (59) / 28 (78)	4.5 (1.1 to 19) *	3.4 (0.55 to 21)	↑	
E. coli counts at 12 mo	28 (100) / 35 (97)	5.3 (1.7 to 17) **	15 (3.6 to 59) †	↑	
Enterococcus counts at 1 w	23 (79) / 23 (72)	462 (40 to >1000) ‡	157 (8.9 to >1000) †	↑	
Enterococcus counts at 2 w	23 (85) / 25 (76)	100 (14 to >100) ‡	53 (6.3 to >100) †	↑	
Enterococcus counts at 1 mo	24 (83) / 32 (89)	51 (5.7 to >100) †	65 (4.6 to >100) **	↑	
Non-*E. coli* counts at 1 w	6 (21) / 3 (9)	716 (0.69 to >1000)	7727 (60 to >10 000) †	↑	
Non-*E. coli* counts at 12 mo	16 (57) / 15 (42)	14 (2.7 to 76) **	3.0 (0.08 to >100)	↑	
Bacteroides counts at 2 w	10 (34) / 13 (36)	27 (1.6 to >100) *	21 (1.4 to >100) *	↑	
CoNS counts at 2 mo	23 (82) / 31 (86)	6.8 (1.0 to 46) *	9.3 (0.91 to 95)	↑	
Bifidobacterum counts at 12 mo	26 (93) / 36 (100)	0.08 (0.01 to 0.59) *	0.02 (0.00 to 2.1)		↓

Statistical analyses were performed using generalized estimating equations (GEE) to account for intra-individual correlation in repeated measures. Results are presented as difference (fold change) in population counts in colonized children of bacterial variables associated with birth order, unadjusted and adjusted for potential confounders, with 95% confidence intervals (CIs). Significant differences between groups are indicated by * $p < 0.05$, ** $p < 0.01$, † $p < 0.001$, ‡ $p < 0.0001$, Wald tests on coefficients obtained from GEE models.

Supplemental Table S6. Bacterial variables with colonization rate associated with delivery mode, unadjusted and adjusted for birth order, exposure to antibiotics during delivery, breastfeeding and infant sex.

		**Percent difference in colonization rate (95% CI) Children cesarean vs vaginally-delivered**			
**Variable and age at sampling**	**n (%) colonized in *sectio* / vaginal**	**Unadjusted**	**Adjusted [a]**	**Higher (↑) / Lower (↓) in *sectio* group**	
E. coli colonization at 3 d	1 (11) / 34 (63)	-45 (-73 to -17) **	-47 (-77 to -18) **		↓
E. coli colonization at 1 w	2 (20) / 35 (69)	-47 (-78 to -17) **	-54 (-85 to -23) †		↓
E. coli colonization at 18 mo	10 (100) / 48 (92)	+8 (0.0 to +15) *	+7 (-29 to +43)	↑	
Non-*E. coli* colonization at 2 mo	8 (80) / 18 (34)	+47 (+16 to +77) **	+56 (+24 to +88) †	↑	
Non-*E. coli* colonization at 4 mo	8 (80) / 24 (44)	+36 (+5 to +66) *	+24 (-26 to +759	↑	
Clostridium colonization at 1 mo	9 (100) / 30 (55)	+47 (+33 to +61) ‡	+58 (+29 to +87) ‡	↑	
Clostridium colonization at 6 mo	10 (100) / 48 (87)	+13 (+4 to +22) **	+8 (-8 to +23)	↑	
C. difficile colonization at 6 mo	3 (30) / 18 (33)	-3 (-37 to +31)	-41 (-75 to -7) *		↓
S. aureus colonization at 6 mo	8 (80) / 24 (44)	+36 (+6 to +67) *	+24 (-18 to +41)	↑	
Bifidobacterium colonization at 4 mo	5 (50) / 49 (91)	-40 (-76 to -5) *	-25 (-71 to +20)		↓
Bifidobacterium colonization at 18 mo	10 (100) / 48 (92)	+7 (0.0 to +15) *	+27 (-40 to +93)	↑	
Bacteroides colonization at 1 w	1 (10) / 26 (51)	-42 (-67 to -17) **	-29 (-61 to +3)		↓
Bacteroides colonization at 2 w	1 (10) / 25 (50)	-41 (-66 to -17) **	-27 (-59 to +6)		↓
Bacteroides colonization at 1 mo	1 (10) / 29 (53)	-43 (-67 to -18) †	-27 (-61 to +7)		↓
Bacteroides colonization at 2 mo	2 (20) / 33 (61)	-42 (-72 to -11) **	-23 (-65 to +20)		↓
Lactobacillus colonization at 2 mo	9 (90) / 34 (63)	+27 (+2 to +51) *	+14 (-35 to +64)	↑	

Statistical analyses were performed using generalized estimating equations (GEE) to account for intra-individual correlation in repeated measures. Results are presented as difference in bacterial colonization rate of bacterial variables associated with delivery mode, unadjusted and adjusted for potential confounders, with 95% confidence intervals (CIs). Significant differences between groups are indicated by * $p < 0.05$, ** $p < 0.01$, † $p < 0.001$, ‡ $p < 0.0001$, Wald tests on coefficients obtained from GEE models.

Supplemental Table S7. Bacterial variables with population counts in colonized children associated with delivery mode, unadjusted and adjusted for birth order, exposure to antibiotics during delivery, breastfeeding and infant sex.

		**Fold change in population counts in colonized children (Fold-change (95% CI)) Children cesarean vs vaginally-delivered**			
**Variable and age at sampling**	**n (%) colonized in *sectio* / vaginal**	**Unadjusted**	**Adjusted**	**Higher (↑) / Lower (↓) in *sectio* group**	
Non-*E. coli* counts at 4 mo	8 (80) / 24 (44)	34 (5.6 to >100) †	31 (2.3 to >100) *	↑	
Clostridium counts at 2 w	7 (70) / 19 (38)	193 (18 to >1000) ‡	67 (1.7 to >1000) *	↑	
Clostridium counts at 2 mo	8 (80) / 32 (59)	5.7 (1.3 to 25) *	3.4 (0.5 to 23)	↑	
Clostridium counts at 12 mo	9 (90) / 52 (96)	0.45 (0.04 to 4.6)	0.09 (0.02 to 0.48) **		↓
Lactobacillus counts at 6 mo	6 (60) / 34 (62)	0.00 (0.00 to 0.66) *	0.07 (0.00 to 44)		↓
Lactobacillus counts at 12 mo	5 (50) / 30 (55)	3.1 (0.01 to >1000)	870 (3.7 to >1000) *	↑	
Ratio anaerobe/facultative at 2 w	-	0.09 (0.02 to 0.53) **	0.19 (0.01 to 4.8)		↓
Ratio anaerobe/facultative at 2 mo	-	0.09 (0.01 to 0.67) *	0.67 (0.02 to 28)		↓
Ratio anaerobe/facultative at 4 mo	-	0.14 (0.02 to 0.82) *	0.13 (0.01 to 1.21)		↓
E. coli counts at 1 w	2 (20) / 35 (67)	3.0 (1.0 to 8.6) *	0.42 (0.00 to >100)		↓
E. coli counts at 2 w	4 (40) / 34 (68)	14 (4.1 to 46) ‡	2.8 (0.12 to 67)	↑	
E. coli counts at 18 mo	10 (100) / 48 (92)	0.03 (0.00 to 0.64) *	0.06 (0.00 to >100)		↓
Enterococcus counts at 1 w	7 (70) / 39 (77)	210 (9.2 to >1000) †	15 (0.94 to >100)	↑	
Enterococcus counts at 2 w	9 (90) / 39 (78)	281 (38 to >1000) ‡	53 (0.44 to >1000)	↑	
Enterococcus counts at 1 mo	9 (90) / 47 (86)	203 (26 to >1000) ‡	59 (0.89 to >1000)	↑	
Enterococcus counts at 6 mo	10 (100) / 55 (100)	0.04 (0.00 to 0.43) **	0.09 (0.00 to 11)		↓
C. difficile counts at 1 mo	1 (10) / 7 (13)	0.01 (0.00 to 0.34) *	- [a]		
Bacteroides counts at 4 mo	6 (60) / 30 (56)	5.7 (1.1 to 28) *	55 (0.04 to >1000)	↑	

Statistical analyses were performed using generalized estimating equations (GEE) to account for intra-individual correlation in repeated measures. Results are presented as difference (fold change) in population counts in colonized children of bacterial variables associated with delivery mode, unadjusted and adjusted for potential confounders, with 95% confidence intervals (CIs). Significant differences between groups are indicated by * $p < 0.05$, ** $p < 0.01$, † $p < 0.001$, ‡ p

< 0.0001, Wald tests on coefficients obtained from GEE models. [a] Adjusted fold change not estimable due to small sample size (small number of colonized children in the exposed group).

Supplemental Table S8. Bacterial variables with colonization rate associated with exposure to antibiotics during delivery, unadjusted and adjusted for birth order, delivery mode, breastfeeding and infant sex.

		Percent difference in colonization rate (95% CI) Exposed vs not exposed to antibiotics during delivery			
**Variable and age at sampling**	**n (%) colonized in exposed / not exposed**	**Unadjusted**	**Adjusted**	**Higher (↑) / Lower (↓) in exposed children**	
Bacteroides colonization at 1 w	1 (10) / 26 (51)	-0.42 (-0.67 to -0.17) **	-0.34 (-0.69 to +0.02)		↓
Bacteroides colonization at 2 w	0 (0) / 26 (51)	-0.43 (-0.65 to -0.22) ‡	-0.33 (-0.66 to -0.01) *		↓
Bacteroides colonization at 1 mo	1 (10) / 29 (53)	-0.43 (-0.67 to -0.18) †	-0.34 (-0.70 to +0.02)		↓
Bacteroides colonization at 2 mo	2 (20) / 33 (61)	-0.42 (-0.72 to -0.11) **	-0.37 (-0.77 to -0.02)		↓
Non-*E. coli* colonization at 2 mo	5 (50) / 21 (40)	+0.11 (-0.26 to +0.48)	-0.35 (-0.68 to -0.01) *		↓
Clostridium colonization at 2 w	7 (78) / 19 (37)	+0.35 (+0.02 to +0.69) *	-0.27 (-0.21 to +0.74)		↓
Clostridium colonization at 1 mo	5 (56) / 34 (62)	-0.04 (-0.43 to +0.35)	-0.35 (-0.69 to -0.01) *		↓
Clostridium colonization at 6 mo	10 (100) / 48 (87)	+0.13 (+0.04 to +0.22) **	+0.07 (-0.07 to +0.21)	↑	
C. difficile colonization at 2 mo	0 (0) / 8 (15)	-0.15 (-0.24 to -0.05) **	-0.22 (-0.42 to -0.03) *		↓
C. difficile colonization at 12 mo	7 (70) / 14 (26)	+0.44 (+0.10 to +0.78) *	+0.32 (-0.44 to +1.09)	↑	
CoNS colonization at 2 months	10 (100) / 44 (82)	+0.18 (+0.08 to +0.29) †	+0.25 (-0.02 to +0.51)	↑	
S. aureus colonization at 6 mo	8 (80) / 24 (44)	+0.36 (+0.06 to +0.67) *	+0.01 (-0.38 to +0.39)	↑	
E. coli colonization at 6 mo	10 (100) / 49 (89)	+0.11 (+0.02 to +0.19) *	+0.30 (-0.04 to +0.64)	↑	
E. coli colonization at 18 mo	10 (100) / 48 (92)	+0.08 (0.00 to +0.15) *	+0.08 (-0.30 to +0.46)	↑	
Bifidobacterium colonization at 1 w	3 (30) / 43 (84)	-0.55 (-0.88 to -0.21) **	-0.35 (-0.78 to +0.07)		↓
Bifidobacterium colonization at 2 w	4 (44) /46 (90)	-0.42 (-0.79 to -0.05) *	-0.28 (-0.74 to +0.17)		↓
Bifidobacterium colonization at 4 mo	5 (50) / 49 (91)	-0.40 (-0.76 to -0.05) *	-0.18 (-0.59 to -0.23)		↓

Statistical analyses were performed using generalized estimating equations (GEE) to account for intra-individual correlation in repeated measures. Results are presented as difference in bacterial colonization rate of bacterial variables associated with exposure to antibiotics during delivery, unadjusted and adjusted for potential confounders, with 95% confidence intervals (CIs). Significant differences between groups are indicated by * $p < 0.05$, ** $p < 0.01$, † $p < 0.001$, ‡ $p < 0.0001$, Wald tests on coefficients obtained from GEE models.

Supplemental Table S9. Bacterial variables with population counts in colonized children associated with exposure to antibiotics during delivery, unadjusted and adjusted for birth order, delivery mode, breastfeeding and infant sex.

		**Fold change of population counts in colonized children (95% CI) Exposed vs not exposed to antibiotics during delivery**			
**Variable and age at sampling**	**n (%) colonized in exposed / not exposed**	**Unadjusted**	**Adjusted**	**Higher (↑) / Lower (↓) in exposed children**	
Ratio anaerobe/facultative at 1 w	-	0.05 (0.01 to 0.37) **	0.06 (0.00 to 0.76) *		↓
Ratio anaerobe/facultative at 2 w	-	0.11 (0.02 to 0.69) *	0.43 (0.02 to 7.7)		↓
Ratio anaerobe/facultative at 4 mo	-	0.10 (0.02 to 0.53) **	0.21 (0.03 to 1.3)		↓
Ratio anaerobe/facultative at 12 mo	-	0.16 (0.04 to 0.75) *	0.83 (0.04 to 16)		↓
S. aureus counts at 1 w	3 (30) / 14 (28)	141 (1.4 to >1000) *	119 (0.31 to >1000)	↑	
S. aureus counts at 2 w	3 (33) / 20 (39)	354 (12 to >1000) †	239 (4.6 to >1000) **	↑	
S. aureus counts at 1 mo	7 (70) / 22 (40)	85 (3.1 to >1000) **	82 (0.37 to >1000)	↑	
S. aureus counts at 4 mo	7 (70) / 23 (43)	55 (12 to 251) ‡	123 (4.8 to >1000) **	↑	
S. aureus counts at 18 mo	4 (40) / 11 (21)	8.0 (1.1 to 61) *	- [a]		
E. coli counts at 1 w	4 (40) / 33 (65)	9.0 (1.7 to 47) *	2.23 (0.25 to 20)	↑	
E. coli counts at 2 w	4 (44) / 34 (67)	21 (6.9 to 65) ‡	5.3 (0.40 to 72)	↑	
E. coli counts at 1 mo	6 (60) / 39 (71)	6.6 (2.0 to 22.14) **	8.1 (0.91 to 72)	↑	
E. coli counts at 4 mo	9 (90) / 48 (89)	9.3 (3.43 to 25) ‡	6.6 (1.6 to 28) *	↑	
E. coli counts at 12 mo	10 (100) / 53 (98)	11 (3.3 to 35) ‡	0.51 (0.06 to 4.5)		↓
Non-E. coli counts at 12 mo	5 (50) / 26 (48)	11 (1.8 to 65) *	4.2 (0.00 to >100)	↑	
Non-E. coli counts at 18 mo	3 (30) / 25 (48)	20 (1.6 to >100) *	- [a]		
Enterococcus counts at 1 w	9 (90) 37 (73)	274 (17 to >100) ‡	5.4 (0.38 to 77)	↑	
Enterococcus counts at 2 w	8 (89) / 40 (78)	95 (6.5 to >100) †	1.7 (0.03 to 97)	↑	
Enterococcus counts at 6 mo	10 (100) / 55 (100)	0.09 (0.01 to 0.54) **	0.18 (0.01 to 3.1)		↓
CoNS counts at 2 w	9 (100) / 51 (100)	12 (2.2 to 68) **	15 (0.92 to 241)	↑	
CoNS counts at 1 mo	7 (70) / 51 (93)	8.3 (1.12 to 61.21) *	20 (0.62 to >100)	↑	

CoNS counts at 4 mo	8 (80) / 46 (85)	34 (1.52 to >100) *	22 (0.13 to >100)	↑	
Bifidobacterium counts at 1 w	3 (30) / 43 (84)	16 (3.34 to 72.34) †	12 (0.68 to >100)	↑	
Bifidobacterium counts at 2 w	4 (44) /46 (90)	3.6 (1.44 to 8.81) **	1.7 (0.58 to 4.76)	↑	
Clostridium counts at 1 w	6 (60) / 23 (45)	35 ((1.92 to >100) *	17 (0.47 to >100)	↑	
Clostridium counts at 2 w	7 (78) / 19 (37)	28 (2.15 to >100) *	4.5 (0.10 to >100)	↑	

Statistical analyses were performed using generalized estimating equations (GEE) to account for intra-individual correlation in repeated measures. Results are presented as difference (fold change) in population counts in colonized children of bacterial variables associated with exposure to antibiotics during delivery, unadjusted and adjusted for potential confounders, with 95% confidence intervals (CIs). Significant differences between groups are indicated by * $p < 0.05$, ** $p < 0.01$, † $p < 0.001$, ‡ $p < 0.0001$, Wald tests on coefficients obtained from GEE models. [a] Adjusted fold change not estimable due to small sample size (small number of colonized children in the exposed group).
